# Effects of Maternal Omega-3 Supplementation, Sex, and Strain on Chick Behaviour During Social Isolation

**DOI:** 10.3390/ani16121852

**Published:** 2026-06-16

**Authors:** Rosie H. Whittle, Elijah G. Kiarie, Tina M. Widowski

**Affiliations:** 1 Department of Animal Biosciences, University of Guelph, Guelph, ON N1G 2W1, Canada; rw114@uark.edu (R.H.W.); ekiarie@uoguelph.ca (E.G.K.); 2 The Campbell Centre for the Study of Animal Welfare, University of Guelph, Guelph, ON N1G 2W1, Canada; 3 Department of Poultry Science, University of Arkansas, Fayetteville, AR 72701, USA

**Keywords:** fear, broiler chicken, layer chicken, maternal diet, omega-3 fatty acids

## Abstract

Omega-3 fatty acids transferred from breeder hens to their developing offspring via the egg may affect the offspring’s emotional reactivity and fearfulness. In one experiment, broiler (meat) parent flocks were fed flaxseed or control diets during rearing, laying, or both. In a second experiment, ISA Brown and Shaver White layer (egg) parent flocks were fed flaxseed or control diets throughout rearing and laying. Male and female broiler offspring and female layer offspring underwent a five-minute social isolation test at 4–6 days of age. Vocalisations, escape attempts, and freezing (immobility) durations were recorded. In the broiler experiment, maternal laying diet and sex interacted to affect vocalisation frequency (χ^2^ = 5.57, *p* = 0.02). Male broiler chicks from the flaxseed maternal laying diet vocalised most frequently (*p* < 0.04). An interaction between the rearing diet, laying diet, and sex affected escape attempts in broiler offspring (χ^2^ = 6.03, *p* = 0.01). Control-control males made the fewest escape attempts. In the layer experiment, no maternal diet effects were observed in any offspring, but Brown chicks vocalised substantially more than White chicks (χ^2^ = 4.56, *p* = 0.03), and White chicks spent more time frozen (χ^2^ = 5.86, *p* = 0.02). We found sex-specific effects of maternal flaxseed diets on measures of anxiety and fear of broiler chickens in social isolation. Genetic strain-dependent effects in layer chickens suggest differing behavioural responses to isolation.

## Simple Summary

Omega-3 fatty acids in breeder hen diets can influence offspring behaviour through the egg. In two separate experiments, we investigated whether flaxseed diets fed to broiler and layer parent flocks during rearing and/or laying affected fear-related behaviours in chicks. In both experiments, broiler and layer chicks were tested in a five-minute social isolation test at 4–6 days of age, recording vocalisations, escape attempts, and freezing. Broiler offspring showed sex-specific responses: males from flaxseed-fed laying hens vocalised most, and diet combinations influenced escape attempts. Layer offspring showed no maternal diet effects, but ISA Brown chicks vocalised more than Shaver White chicks, while White chicks froze for longer. These findings suggest that maternal flaxseed diets can alter fear responses in broilers, and genetic strain influences response to isolation in layers.

## 1. Introduction

Omega-3 fatty acids (n-3 FA), critical for many aspects of peri-natal development, are passed from mother to offspring. In birds, this occurs through the nutritional content of the egg yolk [1]. Shorter-chain n-3 FA, such as alpha-linolenic acid (ALA), are found in plant sources such as flaxseed and chia, whereas longer-chain n-3 FA, like docosahexaenoic acid (DHA), are found in marine sources, including algae and fish oil [2]. Commercial diets for chickens (*Gallus gallus domesticus*) are typically high in omega-6 FA (n-6 FA) and low in n-3 FA due to the use of cost-effective ingredients such as corn and soybean [3]. These diets and subsequent eggs, with a high ratio of n-6 to n-3 FA, may not be adequate for developing chicken embryos. N-6 and n-3 FA require the same enzymes for conversion to longer-chain FA; therefore, an excess of n-6 FA reduces the conversion of n-3 FA [2]. N-3 FA are essential for early brain development, and a deficiency during this critical period may result in long-term adverse effects [4]. For example, rat pups (*Rattus norvegicus domestica*) deficient in n-3 FA during embryonic development have reduced brain size due to the delayed onset of neurogenesis [5]. Changes in the brain due to deficiency or supplementation of n-3 FA can result in behavioural changes, notably emotional reactivity, anxiety, or fearfulness [6]. While anxiety and fear are similar, anxiety typically arises in response to a non-specific or unknown threat, whereas fear is the response to a specific stimulus perceived as a threat [7].

Maternal n-3 FA deficiency in humans has been associated with anxiety and depression [8]. Rodents have often been used to model human anxiety. N-3 FA deficiency in mice (*Mus musculus*) during prenatal development has been shown to trigger hyperactivity of the HPA axis, inactivate glucocorticoid receptor pathways in the prefrontal cortex, and cause dendritic atrophy in brain regions associated with mood disorders in humans [9,10]. However, the effect of n-3 FA deficiency on rodent anxiety may be situation-dependent. Fedorova and Salem [6] found that under stressful conditions, n-3 FA-deficient mice showed higher anxiety during an elevated plus maze; they spent less time in open arms and performed fewer exploratory behaviours than n-3 FA-adequate mice. However, no differences were found between n-3 FA-adequate and deficient mice kept in non-stressful conditions [6]. Postnatal supplementation of n-3 FA can also influence anxiety. Research in grey mouse lemurs (*Microcebus murinus*) fed n-3 FA-supplemented diets for five months found that lemurs showed lower anxiety levels during an open-field test than control animals [11]. Additionally, piglets (*Sus scrofa domesticus*) from mothers supplemented with fish oil during lactation had lower cortisol levels in response to weaning separation than those from non-supplemented mothers [12]. This may be attributed to decreased separation anxiety or fearfulness.

Few studies have investigated the effects of maternal n-3 FA on offspring fear or anxiety in birds; these appear to be limited to commercial poultry and show conflicting evidence. One study in Muscovy ducklings (*Cairina moschata*) showed that ducklings from n-3 FA-fed mothers (plant and marine oils) had shorter tonic immobility than those from control-fed mothers [13], suggesting that maternal n-3 FA decreased offspring fearfulness. However, in the same study, Baéza et al. [13] found no differences in measures of anxiety or fearfulness between maternal n-3 FA ducklings and controls during social isolation and emergence tests. Research in egg-laying chickens showed that chicks from hens with diets supplemented with fish oil had longer tonic immobility durations and greater food neophobia than those from mothers fed a control diet, suggesting that maternal n-3 FA increased fear across several contexts [14]. In another study, maternal-fed n-3 FA had mixed effects on measures of fearfulness in layer chicks. Chicks from laying hens fed diets supplemented with fish oil were more fearful of eating from a novel feeder but were not more fearful of new food types than controls [15]. These few studies highlight that maternal n-3 FA can potentially affect chickens’ fear response, but that it might vary across contexts.

The experiments reported here were part of a larger project examining the effects of maternal supplementation of n-3 FA on the cognition and fearfulness of layer and broiler chickens. We previously found that maternal supplementation of n-3 FA by feeding flaxseed resulted in a larger brain size in broiler chickens and altered n-3 FA concentrations in layer offspring brains [16]. Therefore, there is potential for the offspring’s behaviour to be altered. Here, we aimed to determine the effect of maternal-fed n-3 FA on the response to social isolation in two experiments using the same two chicken models: broiler chickens selected for meat production and layer chickens selected for egg production. For both experiments, we used a social isolation test adapted from Peixoto et al. [17] that has been well-validated as a pharmacological model for anxiety and depression in chickens using anxiolytics [18], cognitive bias tests [19], and a combination of both [20]. For our purposes, we used the first five minutes of the test validated by Sufka and colleagues [18], which assessed the anxiety response of chicks to separation from conspecifics. We selected the first five minutes of the test because we were specifically interested in capturing the anxiety aspect of this test. After five minutes of this isolation test, chicks enter a depression-like state of behavioural inhibition, which was not of interest for our study. Chickens are socially motivated [21,22] and, when separated, attempt to reinstate contact with their conspecifics through vocalisations corresponding to a sensitive period in the first week of life [18,22,23]. Additionally, we recorded freezing behaviour and escape attempts during social isolation, fear behaviours commonly observed in chickens [24,25]. We hypothesised that the maternal n-3 FA diet would alter the number of vocalisations, escape attempts, and the duration of freezing behaviour during the social isolation test.

## 2. Materials and Methods

The University of Guelph Animal Care Committee, following the Canadian Council on Animal Care Guidelines, considered and approved all use of animals and procedures in this study (Animal Utilization Protocol #4246; approved 27 July 2019). All animals were housed at the University of Guelph Arkell Poultry Research Station (Guelph, ON, Canada). A full description of the experimental design and diet formulations can be found in Whittle et al. [26], but they are described briefly below.

### 2.1. Animals and Housing

#### 2.1.1. Broiler Breeder Experiment

This experiment ran from July to December 2019. Ross 708 broiler breeders (213 females, 41 males) were obtained from Aviagen (Aviagen Inc., Huntsville, AL, USA). They were housed in same-sex floor pens (4.28 m^2^), eight female pens, and two male pens, until 18 weeks of age (WoA). At 18 WoA, broiler breeders were moved into adult housing. At 20 WoA, three males were added to each female pen. Broiler breeder hens were fed different maternal rearing (0 to 17 WoA) and laying diet combinations (starting at 18 WoA) of control and n-3 FA-enriched flaxseed diets (LinPRO, O&T Farms Ltd., Regina, SK, Canada) as a source of alpha-linolenic acid (ALA). This 2 × 2 factorial design resulted in four maternal rearing–laying diet combinations: control-control, flaxseed-control, control-flaxseed, and flaxseed-flaxseed. Males were fed a control diet. The control and flaxseed diets were isocaloric and isonitrogenous across all production phases. Inclusion of flaxseed at 2.6%, the inclusion required for omega-3 table egg standards, reduced the n-6:n-3 ratio from ~18–28:1 in control diets to ~3–4:1 in flaxseed diets. Fertile hatching eggs were collected from the broiler breeders at 30 and 33 WoA for incubation, resulting in two cohorts of broiler offspring.

#### 2.1.2. Broiler Offspring

Broiler offspring (480 males, 480 females) were housed in two rooms per cohort, with 12 mixed-sex pens per room (10 males, 10 females per pen). They were housed in floor pens (4.40 m^2^) with wood shavings as litter and fed a commercially available broiler chicken diet.

#### 2.1.3. Layer Breeder Experiment

This experiment ran from June 2020 to September 2021. Commercial layer breeder chickens (384 females, 48 males) were obtained from Hendrix Genetics (Cambridge, ON, Canada) and fed either a control diet or an n-3 FA (ALA)-enriched flaxseed diet (LinPRO, O&T Farms Ltd., Regina, SK, Canada) during rearing and lay. The control and flaxseed diets were isocaloric and isonitrogenous across all production phases. Inclusion of flaxseed at 2.6% reduced the n-6:n-3 ratio from ~12–15:1 in control diets to ~5:1 in flaxseed diets. A white-feathered (Shaver White) and brown-feathered (ISA Brown) strain of layer breeder was used. Four pens of female and one pen of male breeders per diet and strain combination were housed in floor pens with elevated platforms and perches. Males and females were housed separately until 18 weeks of age. Fertile hatching eggs were collected from the layer breeders at 30 and 36 WoA, resulting in two cohorts of layer offspring.

#### 2.1.4. Layer Offspring

Layer offspring were sexed following hatching and 860 females were housed in two rooms per cohort, with 12 identical pens per room (16–22 females per pen). Only female offspring were kept, maintaining external validity with commercial poultry practice. They were raised in furnished floor pens (4.28 m^2^) containing a ramped platform, elevated platform, and perch. They were fed commercially available layer chicken diets.

### 2.2. Social Isolation Tests

In both the broiler and layer experiments, identical methods were used for social isolation tests conducted on offspring at 4–6 days of age. For each broiler maternal diet combination, 36 male and 36 female chicks were tested (N = 288). For each layer strain and maternal diet combination, 46 chicks were tested (N = 184). The number of chicks tested for each experiment was predetermined prior to hatching using a power analysis. Chicks were selected randomly from each pen and marked with livestock paint after the test to avoid retesting. Pens were tested systematically to balance treatments across time of day.

**Figure 1:**
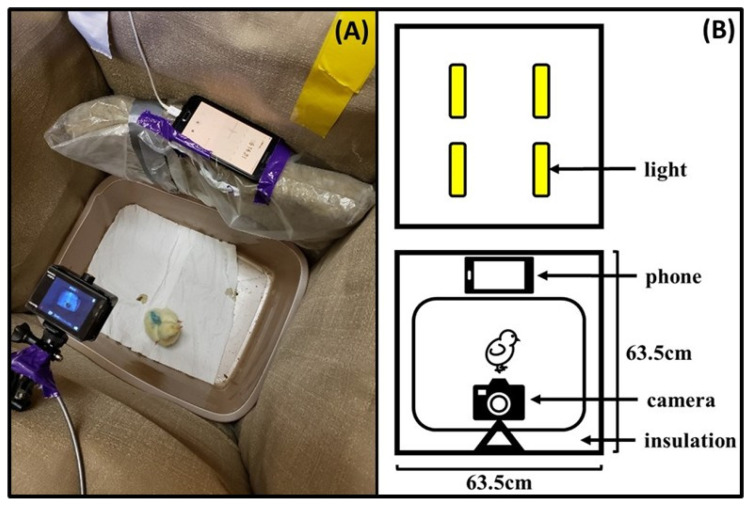
Experimental set-up for social isolation test. (**A**) Experimental set-up with a chick in situ, showing the location of the video camera and voice recorder. (**B**) Labelled overhead diagram of the testing arena showing dimensions and locations of the equipment and starting position of the chick.

The testing arena was a wooden box insulated with acoustic fabric (63.5 cm high × 63.5 cm deep × 63.4 cm wide), providing auditory and visual separation from other chickens and the experimenter. On the inside of the box lid, four LED strip lights were attached to the roof using Velcro. Chicks were carried from their home pen to the testing room and placed inside the box. The recording was started, the lid was shut, and the experimenter left the room. The chick remained in the testing box for five minutes. All tests were video-recorded (4 K Action camera Vision 3, Dragon Touch, Cuauhtémoc, Ciudad de Mexico, Mexico), and voice recordings (Samsung Voice Recorder app v. 23.3.55.16, Suwon-si, Republic of Korea) were taken as a backup. Tests were conducted between 09:00 and 12:00 h and 13:00 and 17:00 h, and chicken strain and maternal diet treatment were balanced across time of day. After testing, all chicks were marked with livestock paint and given a uniquely numbered wing tag to avoid retesting individuals. The testing apparatus is shown in Figure 1.

Videos were analysed using The Observer XT 14 (Noldus, Wageningen, The Netherlands). Vocalisations (all occurrences), escape attempts (all occurrences, classified as jumping upwards, usually preceded by looking up and directed towards the wall of the box), and freezing behaviour (duration, classified as the chick remaining completely immobile for three seconds or more) were recorded.

Intra- and inter-observer reliability was calculated in Microsoft Excel using the Pearson Product-Moment Correlation Coefficient. Tests from the broiler social isolation experiment were coded by three observers, with Observer 1 analysing 60%, Observer 2 30%, and Observer 3 10% of the videos. All observers were blind to maternal diet treatments and the hypothesis, but not sex (male and female chicks were marked with different-coloured livestock paint). Intra-observer reliability was assessed for Observer 1; vocalisations r = 0.99, escape attempts r = 0.99, and freezing duration r = 0.94. The other observers were then correlated with Observer 1 to assess inter-observer reliability. Observer 2; vocalisations r = 0.99, escape attempts r = 0.99, freezing duration r = 0.84. Observer 3; vocalisations r = 0.99, escape attempts r = 0.95, freezing duration r = 0.79. A single observer coded the data from the layer experiment; they were blind to dietary treatment and hypothesis but not the genetic strain of the chicken which could be distinguished by colour of chick, with the intra-observer reliability for vocalisations of r = 0.99, escape attempts r = 0.99, and freezing duration r = 0.97.

### 2.3. Statistical Analyses

Data from the broiler and layer experiments were analysed separately. Analyses were conducted in R (v. 4.3.2) and R Studio (v. 2021.09.01) using the packages “lme4” (v. 1.1-28) and “emmeans” (v. 1.7.1-1). Model residuals were assessed for model fit using a Shapiro–Wilk test and Q-Q and Tukey–Anscombe plots. Random effects that did not contribute to the variation in the data were removed to improve model fit. Significant interactions between fixed main effects were analysed using post hoc Tukey tests with Holm corrections for multiple testing. Means and standard error (SE) were calculated from the raw data.

For the broiler experiment, the maternal rearing diet, maternal laying diet, sex, and their interactions were used as fixed effects. Broiler vocalisation frequency was analysed using linear mixed-effects models (LMM) with offspring pen and day of age as random effects. Broiler escape attempts were analysed using generalised linear mixed effects models (GLMM) using a Poisson distribution; offspring pen nested in cohort and day of age were used as random effects. Freezing duration data of broilers were square-root transformed to improve model fit and analysed using LMM with offspring pen and day of age as random effects.

For the layer experiment, maternal diet, strain, and the interaction between maternal diet and strain were used as fixed effects. Layer offspring vocalisations were analysed using LMM, with cohort and day of age as random effects. The number of escape attempts performed by layer offspring was analysed using GLMM using a Poisson distribution; offspring pen nested in cohort and day of age were used as random effects. Freezing duration of layer offspring during social isolation was square-root transformed to improve model fit and analysed using LMM with cohort and day of age as random effects.

## 3. Results

### 3.1. Broiler Experiment

#### 3.1.1. Vocalisations

**Figure 2:**
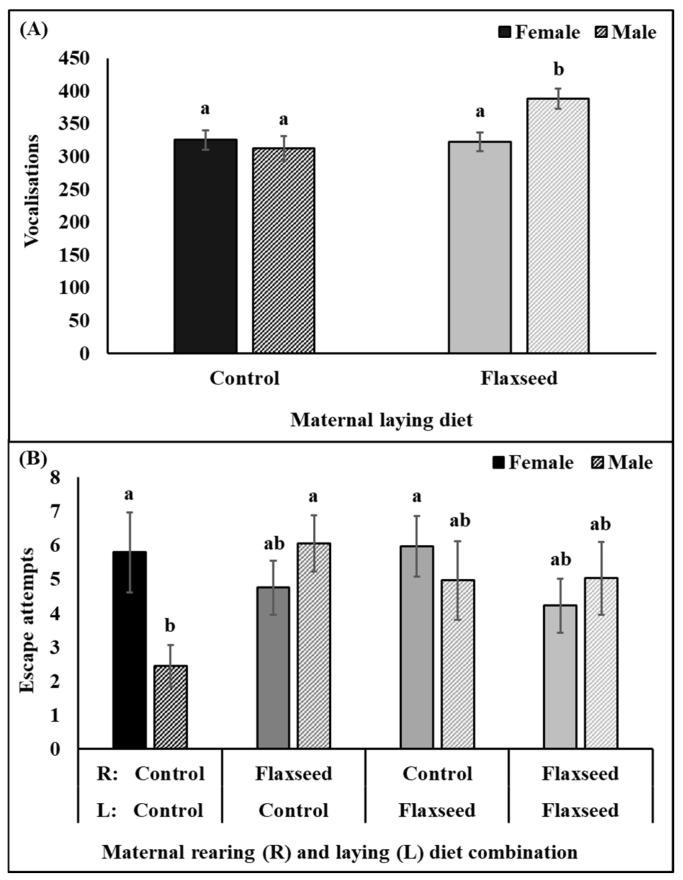
The behaviour of 4–6-day-old broiler chicks during a five-minute social isolation test. (**A**) The average number of vocalisations performed by male and female chicks from broiler breeder hens fed with or without flaxseed during the laying period (χ^2^ = 5.57, *p* = 0.02). (**B**) The average number of escape attempts performed by male and female broiler chicks from broiler breeder hens fed diets with or without flaxseed during the rearing (R) or laying (L) period (χ^2^ = 6.03, *p* = 0.01). Female chicks are depicted with solid bars and male chicks with patterned bars. Error bars show standard error. Significant pairwise comparisons (*p* < 0.05) are shown as different letters.

There was an interaction between maternal laying diet and sex for the number of vocalisations performed by broiler chicks during the social isolation test (Figure 2A, χ^2^ = 5.57, *p* = 0.02). Males from the flaxseed maternal laying diet vocalised more than males from the control maternal laying diet (t = 3.02, *p* = 0.02), females from the flaxseed maternal laying diet (t = 2.91, *p* = 0.02), and females from the control maternal laying diet (t = 2.65, *p* = 0.04). There was no main effect of maternal rearing diet (control 322.0 ± 10.66, flaxseed 351.1 ± 12.36, χ^2^ = 2.37, *p* = 0.13). There were no interactions between maternal rearing diet and maternal laying diet (χ^2^ = 2.34, *p* = 0.13), maternal rearing diet and sex (χ^2^ = 0.97, *p* = 0.32), or maternal rearing diet, maternal laying diet, and sex (χ^2^ = 0.16, *p* = 0.69).

#### 3.1.2. Escape Attempts

There was a 3-way interaction between maternal rearing diet, maternal laying diet, and sex on the number of escape attempts performed by broiler offspring (Figure 2B, χ^2^ = 6.03, *p* = 0.01). Control-control male broilers made significantly fewer escape attempts than control-control females (z = 6.66, *p* < 0.001), control-flaxseed females (z = 3.94, *p* = 0.002), and flaxseed-control males (z = 3.79, *p* = 0.004). Control-control male broilers also tended to make fewer escape attempts than flaxseed-flaxseed males (z = 2.94, *p* = 0.06) and flaxseed-control females (z = 2.91, *p* = 0.07).

#### 3.1.3. Freezing Duration

There was no main effect of maternal rearing diet (control 32.6 ± 4.35, flaxseed 28.4 ± 4.49, χ^2^ = 1.06, *p* = 0.30), maternal laying diet (control 35.3 ± 4.96, flaxseed 25.7 ± 3.74, χ^2^ = 2.06, *p* = 0.15), or sex (male 29.8 ± 4.26, female 31.3 ± 4.58, χ^2^ = 0.19, *p* = 0.66) on the time broiler chicks spent frozen during the test. There were also no significant 2-way interactions between maternal rearing diet and maternal laying diet (χ^2^ = 0.59, *p* = 0.44), maternal rearing diet and sex (χ^2^ = 0.30, *p* = 0.58), and maternal laying diet and sex (χ^2^ = 1.98, *p* = 0.16). There was no 3-way interaction between maternal rearing diet, maternal laying diet, and sex (χ^2^ = 0.12, *p* = 0.73). The mean seconds ± SE spent frozen for each maternal diet combination are control-control 38.94 ± 6.73, control-flaxseed 30.06 ± 7.11, flaxseed-control 26.09 ± 5.28, and flaxseed-flaxseed 26.04 ± 5.40.

### 3.2. Layer Experiment

#### 3.2.1. Vocalisations

**Figure 3:**
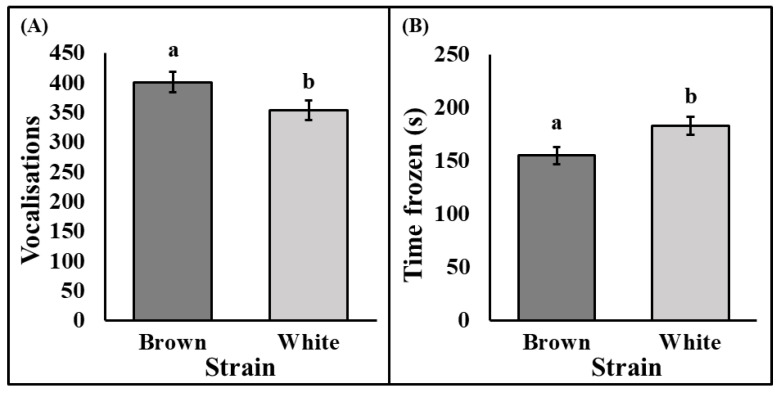
The behaviour of 4–6-day-old laying hen chicks during a five-minute social isolation test. (**A**) The average number of vocalisations performed by ISA Brown and Shaver White chicks (χ^2^ = 4.56, *p* = 0.03). (**B**) The average time spent frozen by ISA Brown and Shaver White chicks (χ^2^ = 5.86, *p* = 0.02). Error bars show standard error. Significant pairwise comparisons (*p* < 0.05) as shown as different letters.

Strain affected the number of vocalisations performed by layer chicks (χ^2^ = 4.56, *p* = 0.03). Brown chicks vocalised more frequently than white chicks when isolated (Figure 3A). However, there was no main effect of maternal diet on the number of vocalisations (control 372.6 ± 17.91, flaxseed 381.2 ± 15.75, χ^2^ = 0.14, *p* = 0.70). There was no interaction between strain and maternal diet (χ^2^ = 0.84, *p* = 0.36). The mean ± SE number of vocalisations for each strain and maternal diet combination was: Brown-control 404.98 ± 25.32, Brown-flaxseed 396.04 ± 23.71, White-control 339.60 ± 24.63, and White-flaxseed 366.57 ± 20.82.

#### 3.2.2. Escape Attempts

There was no main effect of maternal diet (control 9.2 ± 1.17, flaxseed 8.5 ± 0.91, χ^2^ = 0.34, *p* = 0.56), strain (Brown 7.7 ± 0.86, White 10.7 ± 1.18, χ^2^ = 0.75, *p* = 0.39), and there was no interaction between maternal diet and strain (χ^2^ = 0.07, *p* = 0.79) on the number of escape attempts performed by layer chicks during social isolation. The mean ± SE number of escape attempts for each strain and maternal diet combination was: Brown-control 7.83 ± 1.40, Brown-flaxseed 7.48 ± 1.03, White-control 10.69 ± 1.87, and White-flaxseed 9.43 ± 1.49.

#### 3.2.3. Freezing Duration

Strain also affected the duration of freezing during the social isolation test (χ^2^ = 5.86, *p* = 0.02). White chicks spent significantly more time frozen when isolated than Brown chicks (Figure 3B). However, there were no main effects of maternal diet (control 167.3 ± 8.79, flaxseed 171.0 ± 7.79, χ^2^ = 0.33, *p* = 0.56), and no interaction between maternal diet and strain (χ^2^ = 0.65, *p* = 0.42). The mean seconds ± SE spent frozen for each strain and maternal diet combination are as follows: Brown-control 148.91 ± 11.57, Brown-flaxseed 161.54 ± 11.45, White-control 186.00 ± 12.79, and White-flaxseed 180.19 ± 10.53.

## 4. Discussion

This study included two experiments using different avian models from the same species: commercial broiler chickens and commercial egg-laying chickens. These two types of chickens have been divergently selected for different production traits, such as high meat yield and fast growth in broiler chickens or high egg production in egg-laying chickens. These types of chickens differ morphologically, physiologically, and behaviourally; therefore, exploring both models may provide different insights into the effect of maternal-fed flaxseed on offspring behaviour. We explored sex differences only in broiler offspring. In commercial settings, male and female broiler chickens are commonly raised in mixed-sex groups. Therefore, assessing behavioural differences or detecting sex-specific effects of dietary treatments is important for maintaining external validity for commercial broilers. However, for egg-laying chickens, only female offspring are kept for egg production; therefore, we did not include male layer offspring for this study. We included two commonly used, commercially available genetic strains of layer offspring with diverging phenotypes: brown-feathered and white-feathered. These genetic strains are selected from different breeds of chicken; brown-feathered strains are of Rhode Island Red descent, whereas white-feather strains are of White Leghorn descent [27]. Brown- and white-feathered genetic strains of chicken are also known to differ physiologically, morphologically, and behaviourally; therefore, assessing both phenotypes is important [28,29,30], especially given the heritability of traits relating to innate fear response [31].

Proactive (active) and reactive (passive) coping styles are one framework that describes how animals may respond to fear-provoking stimuli. Proactive animals may exhibit more escape attempts, locomotion, and vocalisations in an effort to re-establish social contact with conspecifics. In contrast, reactive individuals may exhibit immobility or freezing behaviour, indicative of behavioural inhibition. These coping styles have been described across species, including great tits [32] and poultry [32,33,34], and have been associated with underlying neuroendocrine regulation and stress reactivity [32,34].

The social isolation test showed sex-specific effects of maternal flaxseed on broiler offspring behaviour. Male broiler chicks from mothers fed flaxseed in their laying diet vocalised more than all other sex-maternal diet combinations. This result suggests that male broiler chicks from the flaxseed-fed mothers may show more anxiety in response to social isolation during this test. In the broiler offspring, we also found a maternal rearing diet, laying diet, and sex interaction in the number of escape attempts. Control-control males performed the fewest escape attempts; this result may indicate that flaxseed maternal diets increased fearfulness in male offspring, resulting in increased escape attempts compared with control-control males. Another explanation for the increased vocalisation and escape attempts in male broiler offspring from flaxseed-fed mothers is that the maternal diet resulted in an alteration of their behavioural response profile towards a more ‘proactive’ stance [33,35], thereby increasing the probability of social reinstatement with conspecifics, rather than passive immobility. These behavioural responses may reflect a shift towards a more proactive copying style in male offspring of flaxseed-supplemented mothers, rather than simply an increase in fearfulness or anxiety per se. However, distinguishing between increased fearfulness and differences in coping strategy is complex. Expanding the behavioural test battery to include additional fear tests (e.g., tonic immobility, novel object, or open field tests) would help to disentangle these interpretations.

Sex differences in maternal fatty acid effects may help explain these patterns. In mice, offspring from mothers fed high n-6 diets show sex-specific differences in fatty acid profiles, despite no differences in total serum or hypothalamic fatty acid content, suggesting that mechanisms of fatty acid accrual may differ between sexes [36]. In rats, increased variability in fatty acid composition in developing male brains under high n-6 maternal conditions may also influence the endocannabinoid system and testosterone production, both of which are implicated in the modulation of behavioural responses [37]. This could help explain why the behavioural differences observed here were limited to male, and not female, broiler offspring from flaxseed-fed breeders.

In the avian literature, it is not uncommon to find sex-specific effects of maternal nutrition on their offspring. Research in zebra finches has shown that when females are nutrient-deprived, their male offspring have increased weight gain and survivability compared to female offspring [38]. It was also found that oxidative stress of zebra finch mothers resulted in heavier male than female offspring [39]. In broiler chickens, the opposite may be true, where female offspring of feed-restricted mothers were heavier than those from ad libitum fed mothers, with no differences found in males [40]. Sex-specific effects on chicken fearfulness in the literature are contradictory and appear to be test-dependent. In one study, male Lohmann Selected Lite chickens were more fearful than females in a tonic immobility test, taking longer to righten after tonic immobility induction [25]. Similarly, female Shaver 288, Thornber 909, and J-line chicks were less fearful in an open field test than male chicks [41]. Other studies have found that female layers are more fearful, perform more distress vocalisations during social isolation [17,25], and take longer to emerge into a novel arena and approach a human [29]. Additionally, females may be more likely to approach familiar chicks, and males are more likely to approach unfamiliar chicks [42]. However, these sex differences in response to fear tests were found in layers and might not be translational for broiler chickens.

While the maternal flaxseed diet did not affect the behaviour of layer offspring, there were differences in behavioural response to social isolation between white and brown-layer offspring. Brown offspring vocalised more, and White offspring spent more time frozen, indicating different responses to the same stimulus. Increased vocalisations in Brown offspring may indicate a more proactive anxiety response to social isolation than the more reactive fear response of freezing behaviour observed in White offspring. The results of our study also support literature showing that brown-feathered chicks vocalise more than white-feathered chicks during a social isolation test [17] or an open-field test [43]. However, other research looking at fearfulness has found opposing effects. For example, Hocking et al. [30] found that white-feathered hens vocalised more frequently during an emergence test, whereas brown-feathered hens had a longer latency to walk. Similarly, Hymel et al. [44] found that commercial white-feathered chicks vocalised more than brown-feathered chicks during the first three minutes of isolation. Care must be taken when interpreting fearfulness in different strains of chickens because they may be similarly fearful but exhibit different fear response profiles (reactive vs. proactive) as highlighted by Rentsch et al. [45] in a meta-analysis, which suggested that the type of fear test affected the fear response of brown- and white-feathered laying hen pullets.

The changes in broiler offspring behavioural responses to social isolation correspond with our previous findings published elsewhere from the same experiments [16]. We found that broiler breeders fed without flaxseed produced eggs with higher n-6 FA to n-3 FA ratios, resulting in broiler offspring with smaller brain-to-body weight ratios than those of broiler breeders fed flaxseed diets. It is important to note that the brains were from unsexed day-old chicks, so it is uncertain whether there were sex-specific effects on brain size. The maternal diet only affected broiler offspring and not layer offspring behaviour. This divergence suggests that broilers and layers may differ in how maternal fatty acid availability influences neurodevelopment and subsequent behaviour. This finding corresponds with our previously published data from the same experiments, in which we found that maternal flaxseed diets increased the brain size of broiler, but not layer, offspring [16]. In contrast, the brain fatty acid profile was altered in layers but not in broilers [16]. These differences may, in part, reflect broader genetic and selection-related divergence between broiler and layer chickens. Domestication has been shown to substantially affect stress responses; for example, domesticated white laying hens exhibit attenuated stress responses compared to their wild ancestors, the red junglefowl [46,47]. Furthermore, broiler and layer lines have diverged considerably through selection, with brown layers being more genetically similar to broilers than white layers [27]. Although direct comparisons between broiler and layer behaviour are limited, behavioural differences between brown and white layer strains are well documented [17,48], and variation also exists within white-feathered genetic lines [48]. Together, this highlights that even relatively subtle genetic differences can lead to distinct behavioural phenotypes, likely reflecting differing selection pressures on production, growth, and coping traits.

This dissociation between neurochemical changes and behavioural outcomes suggests that alterations in brain fatty acid composition in layer offspring, observed in the same population of birds in the current study, may not translate into measurable behavioural differences in this context. Alternatively, the differences in fatty acid composition found in layer chicks may influence neural processes not captured by the social isolation test. This highlights the importance of using a broader suite of behavioural tests to explore any consequences of neurobiological changes. Further work could also assess the behaviour of the breeder hens in conjunction with the offspring to assess transgenerational shifts in coping strategies and provide further insight into potential mechanisms underlying maternal dietary effects.

## 5. Conclusions

In conclusion, maternally fed flaxseed produced sex- and strain-specific effects, influencing behavioural responses to social isolation in male broiler offspring only, with no detectable effects in female broilers or layer strains. These findings suggest that maternal diet may shape offspring behavioural responses in a context-dependent manner. This study also highlighted the importance of measuring multiple fear-related behaviours, such as freezing and vocalisations. Chickens of different phylogenetic origins (brown- or white-feathered) may be equally fearful of a stimulus but exhibit different behavioural responses. However, interpretations are limited by the use of a single behaviour test, and further work is required to determine whether these effects reflect changes in fearfulness, anxiety, or coping strategy.

## Data Availability

The raw data and associated models are openly available at Borealis Data Repository (https://doi.org/10.5683/SP3/QM5CR8).
